# Ten simple rules for engaging with artificial intelligence in biomedicine

**DOI:** 10.1371/journal.pcbi.1008531

**Published:** 2021-02-11

**Authors:** Avni Malik, Paranjay Patel, Lubaina Ehsan, Shan Guleria, Thomas Hartka, Sodiq Adewole, Sana Syed

**Affiliations:** 1 College of Arts and Sciences, University of Virginia, Charlottesville, Virginia, United States of America; 2 School of Medicine, University of Virginia, Charlottesville, Virginia, United States of America; 3 Department of Pediatrics, Division of Gastroenterology, Hepatology, and Nutrition, School of Medicine, University of Virginia, Charlottesville, Virginia, United States of America; 4 Department of Emergency Medicine, School of Medicine, University of Virginia, Charlottesville, Virginia, United States of America; 5 Department of Systems and Information Engineering, School of Data Science, University of Virginia, Charlottesville, Virginia, United States of America; Dassault Systemes BIOVIA, UNITED STATES

## Introduction

The first industrial revolution led to mechanical production and steam power; the second, mass production and electrical power; and the third, electronics and computers. Today, as most sectors of the world move forward into the fourth industrial revolution, one centered around data and artificial intelligence (AI), biomedicine finds itself still in the third, lagging behind the rest [1]. Only recently, the exponential growth of technology has facilitated the widespread integration of computers into the biomedical domain from electronization of medical data analysis to automated detection of biomedical images [2–3].

Rather than merely automating time-consuming processes within healthcare, AI stands to reduce medical errors, expand upon the relationships between basic science and clinical medicine, and improve our analysis of existing datasets too large and complex for traditional statistics [3]. Despite these potential benefits, many biomedical facilities are hesitant to incorporate such systems into their practices due to the liability associated with AI making decisions that impact the health of patients [4], such as misdiagnosis (see Rule 8). Additionally, there exists a computational “black box,” a phenomenon describing the difficulty of understanding how AI algorithms arrive at a particular result (see Rule 3). Without a clear means of understanding how these machines generate their output, biomedical facilities are often skeptical of incorporating these “black boxes” into their work practices. As such, the “explainability” issue is an important barrier to overcome before applying these powerful technologies in biomedicine [5].

The lack of understanding around AI and the tantalizing benefits of this new wave of technology suggest the need for professionals in biomedical fields to acquire a basic understanding of AI and its medical applications in order to understand its clinical utility and engage with cutting-edge research. As such, there is a clear need for literature that explains AI in a way that is digestible to professionals in other fields [5]. Without a fundamental understanding of data science models and AI methods, modern biomedical experts who are not well versed in these fundamentals may be intimidated. Introduction to the basics of AI, such as big data analysis, data mining, machine and deep learning, and computer vision, would allow for the expansion of innovative designs in biomedicine.

The importance of biomedical involvement in emerging technology is highlighted in the flaws of contemporary electronic medical records (EMR) that are widely used across the healthcare system. The ideal AI adaptation of EMR would be able to facilitate patient care through a variety of features like tracking changes in medical history of a patient and alerting caretakers of concerning health patterns; however, with the current state of EMR, tasks as simple as sharing medical records between healthcare facilities are burdensome. Though it has many virtues, most biomedical professionals agree that the current implementation of EMR is less than ideal, in part because it was developed and implemented with minimal consideration for the flow of information in the biomedical field [6]. The current weaknesses of EMR should serve as a warning, illustrating the importance of biomedical involvement in the deployment of new technologies. When these advancements inevitably make it to the forefront of clinical medicine, biomedical professionals should feel like they are in the driver’s seat rather than helpless passengers along for the ride. We propose the following rules to allow biomedical professionals to attain some measure of control and strap down their panic at the sight of words such as “algorithms,” “AI,” “machine learning” and the like.

### Rule 1: Don’t panic

Computation-based technologies are ubiquitous in our lives, touching almost every facet of our day-to-day interactions. Nevertheless, the vast majority of us do not understand how these systems operate, let alone how to troubleshoot them when problems become apparent. Quickly overwhelmed and frustrated by error messages, constant update reminders, and pop-up advertisements, many of us have an adverse reaction to the increased incorporation of technology into our daily lives.

As healthcare begins to adopt a new language unfamiliar to most people, there will be pushback. When there are words that we do not understand, such as “machine learning,” our immediate response is to experience an internal error message and shut down. While it is only natural to have an uneasy and uncomfortable feeling when approaching anything unfamiliar, this sensation can be debilitating and prevent the exploration of the unknown. Now is the time to resist the urge to fall back into something comfortable and learn how to embrace that feeling to allow yourself to grow from new experiences.

It should be reassuring that, although many of the terms used in AI seem exotic, they are often deviations on reasonably simple statistical concepts that many biomedical professionals already understand. For example, “a multivariate predictive model using three knots of nonlinearity for continuous values” is fundamentally a linear regression model with some extra bells and whistles. “Deep learning” is a specific method to train neural networks, which are based upon different layers of computational “neurons” that recognize patterns (see Rule 3), much like neurons in the brain firing in response to specific visual inputs [7]. In learning about these new techniques, biomedical professionals will find that they are already familiar with many of the underlying algorithms.

Your preexisting knowledge of statistics will serve as the foundation for your understanding of AI, because AI builds on statistics. Both statistics and AI manipulate data with similar algorithms and differ only in the purpose—inference versus prediction, respectively.

### Rule 2: Don’t feel threatened by an impending “AI takeover”

While technology is advancing rapidly, experts believe we are still light years away from the Hollywood depictions of AI with robots taking over the world. Furthermore, these inaccurate representations of “intelligence” (see Rule 3) only further misunderstanding and induce the fear that robot takeover is inevitable and imminent.

Upon discussion of the integration of more machines into healthcare, many biomedical experts are intimidated by the thought that they are being replaced by robots. The concern is that once machines are able to perform tasks normally done by people, people’s jobs will become unnecessary and obsolete. However, it is important to keep in mind that machines can reach erroneous conclusions (see Rule 8), often because they lack the ability to view a problem from multiple perspectives, ranging from the cell level to the patient and even global level. The “art” of biomedicine reflects this synthesis of data from a variety of sources with the hopes and wishes of a human patient in order to create an action plan. AI in our lifetimes will never achieve the level of biomedical artistry that a human can. Do not think of machines as replacements, but rather one more tool on your toolbelt.

Biomedical imaging is a field which has seen significant change since the incorporation of AI. New image analysis software has facilitated the identification of cells and the diagnosis of conditions. The practice of radiology, for example, has increased in efficiency due to technologies that act as an extra set of eyes (see Rule 6). This would help serve much needed gaps, such as providing AI-driven diagnostic aid systems for residents and fellows in overnight shifts when there is typically less attending supervision for imaging interpretation or in triaging patient investigations with critical findings that need immediate human review. Many have fallen prey to the notion that these programs reduce the need for radiologists; however, there is still the need for radiologists to confirm a software’s computational output and place the findings in context of the whole patient. Artificially intelligent machines are not replacing radiologists; rather, radiologists who understand these machines are replacing radiologists who do not [8].

Some theorists in AI make the distinction between prediction and judgment [9]. Prediction is simply the process of determining the probability of an event. It is becoming cheap, and computers may outperform humans in this area, but it only encompasses part of the complexities of providing medical care. Judgment involves weighing the risk and benefits in the context of an individual to choose a course of action. An algorithm may be able to determine the likelihood that a lesion on a radiograph is malignant, but selecting the best next steps in the context of the patient’s understanding, beliefs, and desires is a process that will likely never be automated.

### Rule 3: Understand what artificial intelligence is

**Fig 1:**
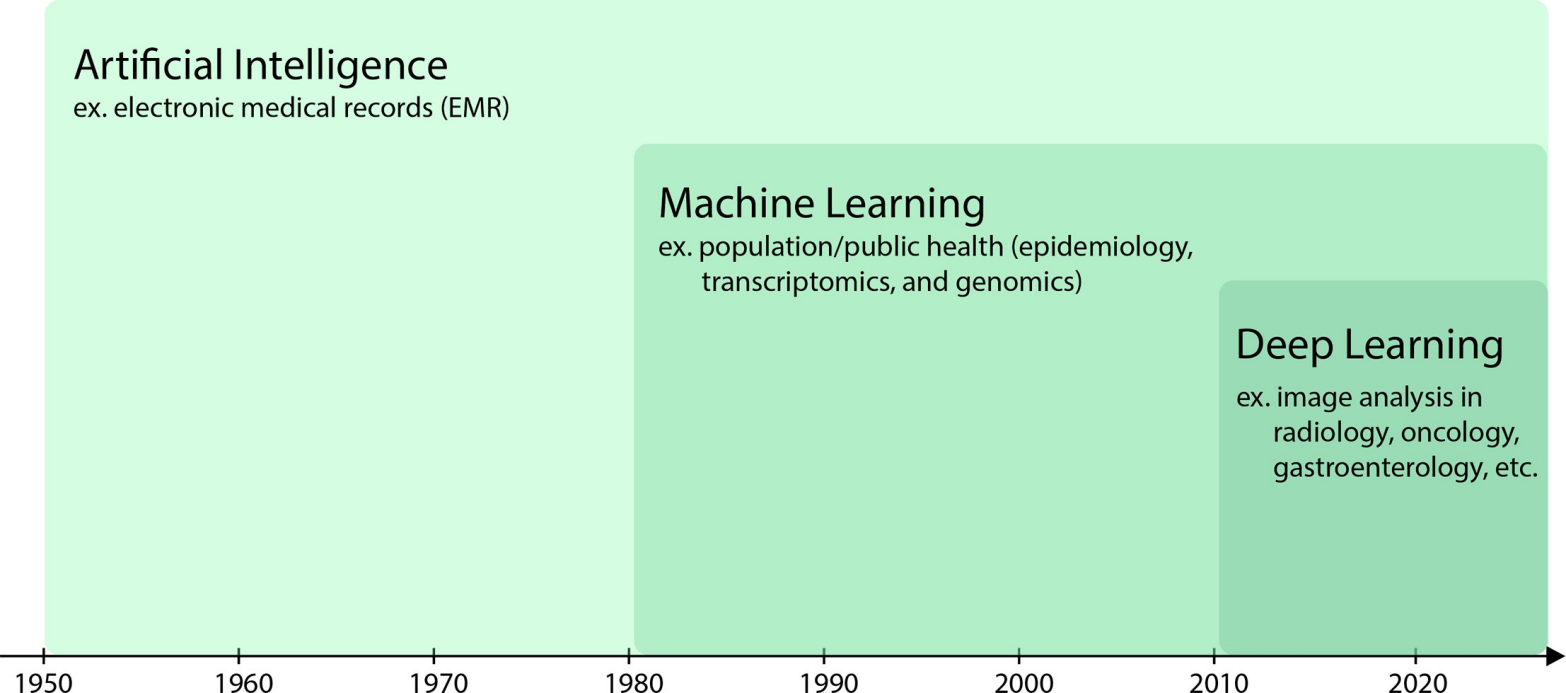
Understand what Artificial Intelligence is. Data analysis techniques have become more advanced over time. The elaboration of artificial intelligence techniques influenced machine learning. Similarly, the sophistication of machine learning prompted evolution into deep learning as shown in this figure adapted from Le Berre and colleagues [13]. Each approach shows an example of its application in the biomedical field.

Artificial intelligence is a term loosely used to describe the ability of designed intelligent agents to mimic human decision-making processes to achieve a desired goal [10–12]. An extension of statistics and data analysis, AI can be programmed to reason and perform tasks in different environments. In this paper, we generally refer to AI in the context of computational data analysis and learning systems that can make predictions from structured (tabular data like patient records) and unstructured data (biomedical images and videos). The term “AI” is frequently confused to be synonymous with “machine learning” and “deep learning,” but they are actually subsets of one another (Fig 1).

Machine learning (ML) is a subfield of study which aims to approximate a mapping function between the input and output from given training data without explicit programming in order to make predictions on new data. Training data are a group of data points for the algorithm to learn patterns and then subsequently make inferences linked to the outcome of interest. The validation data are used to tune the hyperparameters (configuration) of the model by iteratively testing the model’s performance on this set while changing the values of hyperparameters to eventually arrive at the optimal model configuration values. After deriving an optimized model, new observations can be made about the relationship between the data and the outcome. This learning process is essential to produce the predictive behavior of a model for new data and ensure the generalization ability of the learned mapping function. The introduction of new data in the form of a test set then assesses the ML model’s performance after the learning phase.

With the tremendous increases in computing power, the last couple decades saw a rise in the use of neural networks. These networks, loosely modeled on the functioning of the brain, are made up of a network of interconnected units called “neurons.” When given an input, each neuron performs a relatively simple function and outputs either a high value or a very low value. An arbitrarily large number of such units when connected together can approximate complex mathematical functions to map the input to an output. This field of study within ML came to be known as deep learning (DL). Therefore, in recent years, ML generally refers to more traditionally used statistical algorithms to learn the mapping function, while DL refers to the use of neural network architectures to learn the mapping [14].

ML and DL can be easily confused, because they are very similar approaches. For example, an ML model would be given training data with biomedical images of cells and the corresponding classifications of the cells in order to determine what distinct microscope features are linked to specific cell types. A DL model would be given training data with just biomedical images of cells in order to determine the underlying patterns between the microscope features and specific cells, as well as the classifications of the cells. A properly trained DL algorithm would also be able to distinguish more sophisticated features, giving a higher classification accuracy [15].

**Fig 2:**
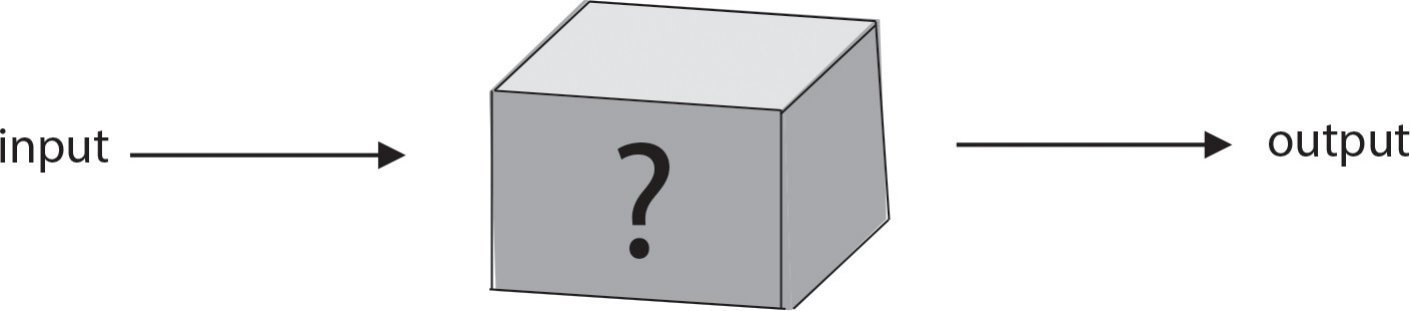
Understand what Artificial Intelligence is. The predictive behaviors of AI models can sometimes be hidden from programmers, who must then view the internal workings as a “black box,” analyzing them based on their inputs and outputs.

You may encounter the term “black box” when looking into AI technology. Traditional computer models are programmed explicitly to perform specific tasks, and their programmers therefore know exactly how they arrive at their end decision. This is not the case with AI models, which are programmed to learn without specific input from humans. As a result, programmers can visualize the inputs and outputs of an AI model, but it is very difficult to explore the process by which the model achieves its decision-making (Fig 2). This lack of “explainability” is what is referred to as the “black box” of AI [16].

This theory of a “black box” has also been generalized and applied outside of the scope of AI to other studies such as philosophy and biomedical sciences. For example, patients can be viewed as the ultimate “black box,” as we often cannot directly observe the internal physiological function of the body [17]. We can try different treatments or therapies and assess for a response to infer internal processes causing a condition. This “black box” visualization has been further extended in the biomedical domain through image analysis (see Rule 5).

### Rule 4: Keep a working dictionary of AI vocabulary

If you have managed to make it past the minefield of unfamiliar words above, congratulations! Many people do not understand that AI is all around us in our everyday lives, from the voice recognition used by home devices such as the Amazon Echo and the image recognition utilized by Facebook to suggest picture tags. An understanding of these words will open doors for you as technology becomes inevitably more incorporated in biomedicine.

Use your new knowledge to seek out more literature about AI. Research the biomedical advancements being made, and understand where AI is already applied in your specialty. Try to relate new ML methods to concepts you already understand. Keep record of the new terms and methods you learn about in order to better your own practice and provide patients the best care possible.

### Rule 5: Try to peek into the “black box” where you can

As mentioned above (see Rule 3), sometimes it is not always clear how computational (or AI) models make decisions. Do not let this discourage you from trying to understand the “black box” where you can. Understanding the basic concepts on which these models are built can help to demystify their inner workings. In AI literature, especially in papers regarding image analysis, this can be done by seeking out studies related to “Explainable AI.” In order to be able to use AI algorithms as tools, biomedical professionals must understand what processes are used to produce the outputs. Algorithms can handle the same problem very differently and generate widely varying results; therefore, understanding their inner workings can help biomedical professionals distinguish which decision-making algorithm is the most appropriate for a given situation. After being verified for validity by a biomedical expert, the program output can then be used as supportive evidence in analysis.

**Fig 3:**
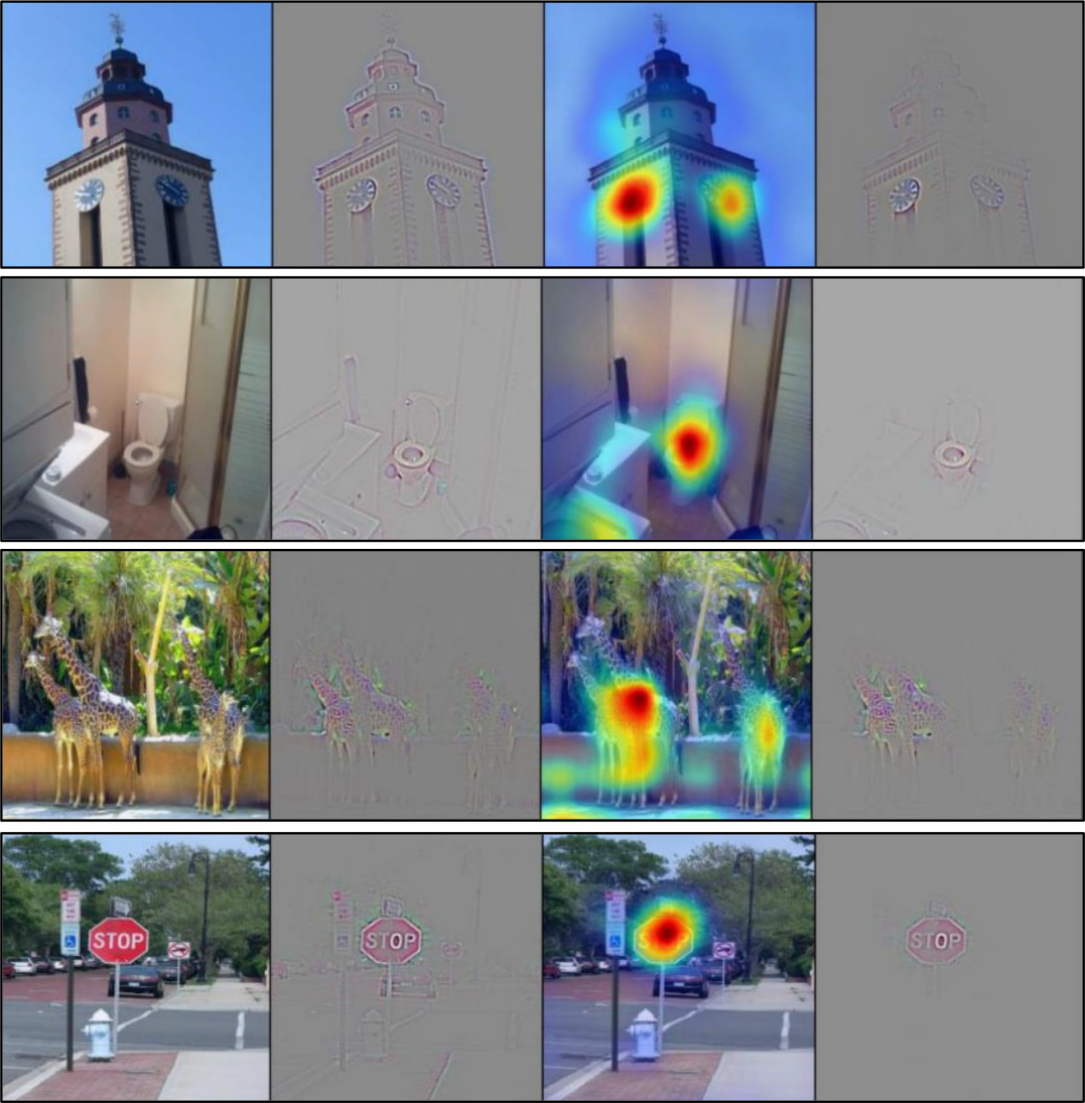
Try to peek into the “black box” where you can. The recognition of objects in images done by Grad-CAMs relies on classification of these objects by programmers. Without the explicit knowledge of what “clocks,” “toilets and sinks,” giraffes, or “stop signs” are, the programmer would not be able to verify the outputs of the Grad-CAM visualization. Mastery of the topic of interest is crucial in the development of such models [19].

**Fig 4:**
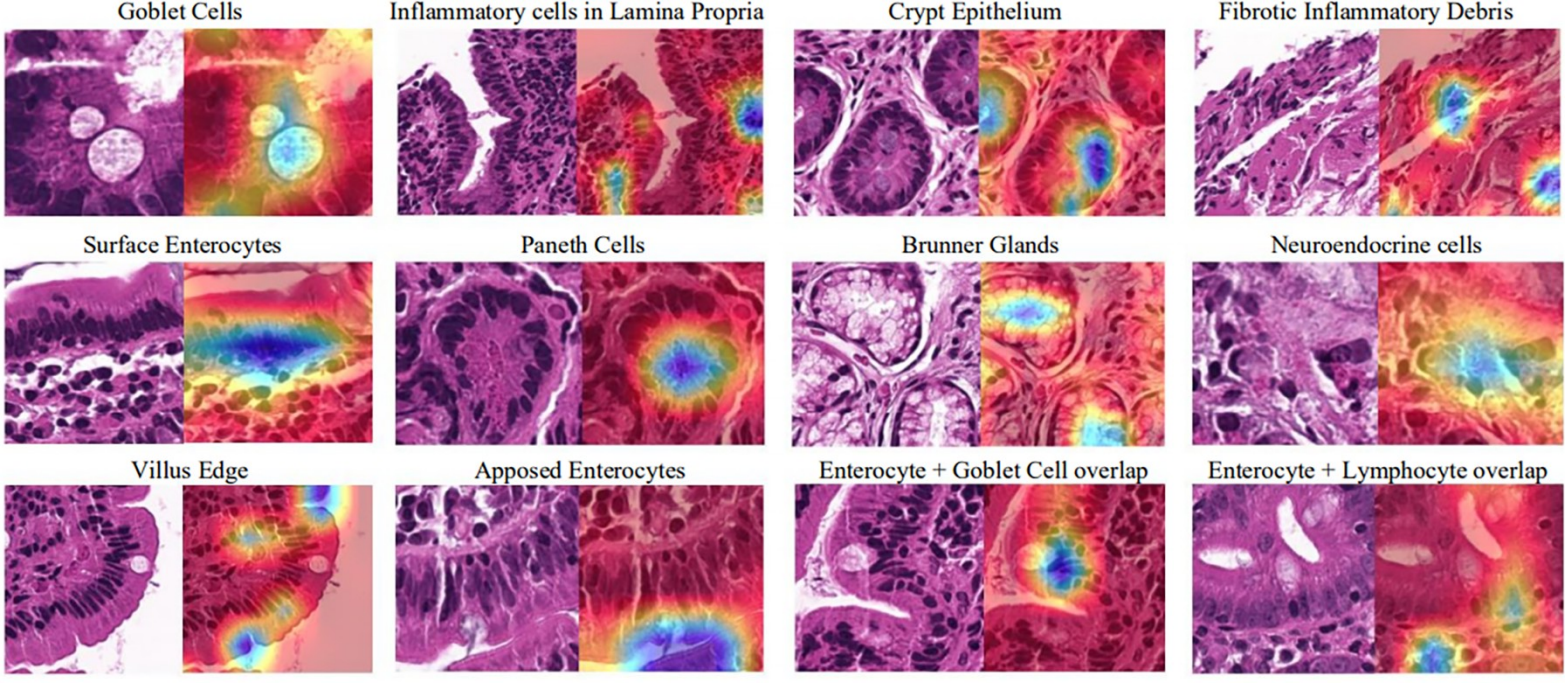
Try to peek into the “black box” where you can. With the help of experts in gastrointestinal tissues, Grad-CAMs can be trained to recognize many different key features (specified by the blue color) in biomedical images to aid with celiac disease classification [20].

The notion of a “black box” has become a significant hurdle preventing AI adaptation into biomedicine through the misconception that it cannot be understood. However, as is done with other advanced biomedical technologies, the results can be interpreted in many ways [18]. Professionals in biomedicine can help find ways to open the “black box” where AI experts are unable to provide insight into how analysis is done and decisions are made. In the development of one such AI approach called Gradient-weighted Class Activation Mapping (Grad-CAM), biomedical experts can review which aspects of an image an AI model uses to make its decision (Fig 3). Even if you do not understand the computational aspect of the biomedical technology in question, you are an invaluable resource for the engineers developing it because only a biomedical expert can make sense of the model’s output (Fig 4).

### Rule 6: Find the potential of AI in your practice

The purposes of integrating these models into biomedicine are to allow for early and efficient diagnosis, to develop personalized medical therapy for patients, and to enhance healthcare operations for the well-being of patients. Biomedical experts come to the table with a wealth of knowledge in fields such as genomics and proteomics, bioimaging, medical imaging, brain and body machine interface, and public and medical health management. This expertise, combined with the firsthand experience of everyday frustrations and struggles that prevent progress in biomedicine, will facilitate the assimilation of helpful AI models into the healthcare space [3].

Years of training in the fact-based sciences make biomedical professionals fluent in convergent thinking. However, we urge you to analyze the problems you face in your particular branch of biomedicine and partake in explorative divergent thinking in order to come up with creative and innovative solutions. Think about the limitations in your practice, issues with current technological methods, and other potential benefits of incorporating new machines.

**Fig 5:**
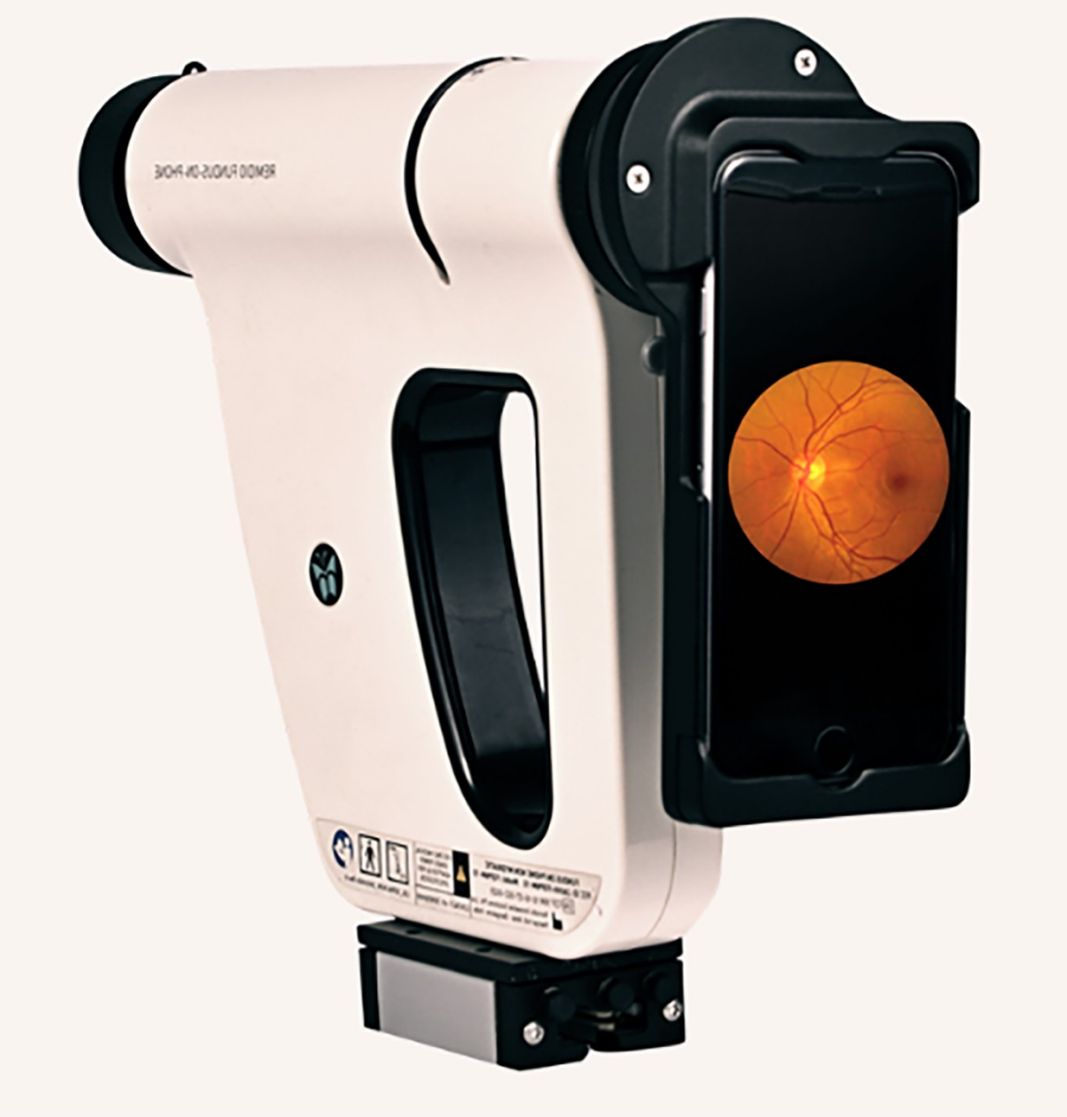
Find the potential of AI in practice. The Remidio “Fundus on Phone” has reformed fundoscopy by creating more accessible and economical technology to optimize healthcare [22].

Recent years have seen rapid growth in the distribution of published papers that use AI methods in biomedical fields, especially bio- and medical imaging [3]. One practical example of the power of technology in biomedicine is the automation of diabetic retinopathy detection using smartphone-based fundoscopy. The combined forces of AI experts, biomedical engineers, authorities in retinal imaging, and physicians have allowed for the development of AI software in Remidio “Fundus On Phone” (Fig 5), which, with a high sensitivity for detecting diabetic retinopathy, has proven to be an effective initial mass retinal screening tool. The handheld device offers convenience by eliminating the need for mydriasis (dilation of the pupil) and allowing primary care physicians to track the progress of the condition [21]. The benefits extend not only to the reduction of ophthalmology referrals and ophthalmologist workload but also to the minimization of healthcare and insurance costs for the patient.

### Rule 7: Partner with AI experts to advance your field

Rest assured, once you have an idea to incorporate AI into your work, you do not have to do it alone. The development of AI models almost always involves a team of researchers with different strengths. Once you have determined where AI can improve your practice, coordinate with experts who understand the possibilities and limitations of technology. With your existing knowledge of the twists and turns of the problem and its context, act as a guide through the process of developing new technology to help yield the most successful outcomes.

Many biomedical professionals can be unsettled and feel as though they are not in the driver’s seat when it comes to the application of “intelligent” technology in their respective fields. Although learning the raw AI algorithms and programming is by far the most challenging part (see Rule 9 for resources to get you started), the more you are familiar with the principles of AI, the better you can facilitate application in your field of expertise. The most fruitful partnerships with data scientists and computer programmers result from multidisciplinary approaches in clinical and lab settings.

### Rule 8: Recognize the ethical implications of AI in biomedicine

The ethical issues related to the use of AI in our lives have been well documented with several contemporary books devoted to explaining this in layman language to the general audience [23–25].

The benefits of AI in biomedicine, while innumerable, are not widely applied due to the liabilities of computational error. With the concern of who is at fault when a machine malfunctions, biomedical facilities are hesitant to integrate such technologies into healthcare applications. The implementation of these systems requires the awareness of biomedical professionals about the decision-making algorithms to prevent harmful consequences for patients.

Eliminating bias is important throughout applications of AI, and underrepresented groups are often at increased risk of the harmful effects of bias. The data that a healthcare program uses must be representative of a population to minimize risks for patients. The collection of this data also increases privacy and consent challenges, which have been especially emphasized in genomics research.

Personal genomics analysis that is offered by the likes of 23andMe and Ancestry have become increasingly popular for their ability to use AI to predict ethnicity and genetic predisposition to diseases like breast cancer and Alzheimer disease. However, with the increasing ability to identify even anonymized genetic data, not only can an individual’s health-related information be exploited by insurance companies, who increase rates for people at risk of certain medical conditions, but also their family members. While recent laws have banned these malpractices, they do not account for loopholes [26].

The National Institutes of Health has addressed many of the concerns that have risen with the expansion of AI in biomedicine [27]; however, because the field of AI is constantly growing and evolving, we recommend that you stay up to date with policies and legislation regarding biomedical ethics.

### Rule 9: Help educate your peers about the relevance of AI

While you might be as excited as we are about the possibilities of AI in the biomedical field, not all of your colleagues will share your enthusiasm. As many biomedical engineers may have experienced already, these AI models are more apt to streamline the healthcare system if there is a welcoming environment for their incorporation. This can be achieved by introducing the ideas and methods from literature or practice to other biomedical professionals. Once others are excited about the potential benefits, the incorporation of biomedical technologies will have much less resistance.

Many institutions have recognized the expanding sector of AI in biomedicine and are developing interdisciplinary programs and courses geared toward current and future technological advancements [28]. AI and data science courses are also offered to physicians as Continuing Medical Education and through undergraduate medical education as supplemental learning to prepare future healthcare workers [29–32]. Take advantage of these resources around you and share them with your peers.

### Rule 10: Remember, AI is not magic

Science fiction writer Arthur C. Clark famously stated that “any sufficiently advanced technology is indistinguishable from magic [33].” It is easy to get carried away under the impression that technology is advancing at tremendous rates and that these models cannot be understood by anyone who is not part of the AI avant-garde. However, the fundamental concepts of most AI techniques are often fairly simple. While implementing these models requires in-depth experience and training, a general understanding of what is happening under the hood is attainable with a reasonably small investment of time.

Even after years of hard work to develop an AI machine, the program can still have many flaws. IBM Watson’s supercomputer was found to often suggest unsafe and incorrect treatment for cancer. Regardless of the mass quantity of data used during development, the AI was not able to provide accurate solutions. With the help of medical specialists, these flaws were identified and the promotion of the product to hospitals and physicians was promptly halted [34].

Understanding AI is simple at its core, and you will realize that its “wizardry” relies on data and programming. AI cannot do everything, and we need to have reasonable expectations of what it can do. Developing and perfecting models into usable tools takes years of hard work, trial and error, and time. Despite this, the benefits are reaped when these artificially intelligent models help humans live happier, healthier lives.

## Conclusion

Congratulations! You have ventured this far into understanding the incorporation of AI in biomedicine. While it is invaluable to have an understanding of the current applications of AI in biomedicine, the development of future technology will require input from biomedical professionals to be successful. AI is a team sport, so we encourage collaboration across fields in order to help transition biomedicine from the third to the fourth industrial revolution. Use the rules listed as a guide from which to build your knowledge. Take it step-by-step, and become a part of the future of biomedicine!

## References

[1] Topol E. Deep medicine: how artificial intelligence can make healthcare human again. New York, New York: Basic Books; 2019.

[2] Smith TM. 10 ways health care AI could transform primary care. American Medical Association 2020 [cited 2020 May 17]. Available from: https://www.ama-assn.org/practice-management/digital/10-ways-health-care-ai-could-transform-primary-care.

[3] Zemouri R, Zerhouni N, Racoceanu D. Deep Learning in the biomedical applications: recent and future status. Appl Sci. 2019 4;9 10.3390/app9081526

[4] Nicholson W. Risks and remedies for artificial intelligence in health care. Brookings. Brookings; 2020 [cited 2020 May 17]. Available from: https://www.brookings.edu/research/risks-and-remedies-for-artificial-intelligence-in-health-care/.

[5] News Center. Stanford Medicine launches health care trends report. News Center. 2017 [cited 2020 May 17]. Available from: http://med.stanford.edu/news/all-news/2017/06/stanford-medicine-launches-health-care-trends-report.html.

[6] Reisman M. EHRs: the challenge of making electronic data usable and interoperable P & T: a peer-reviewed journal for formulary management. MediMedia USA, Inc.; 2017. PMC5565131 28890644

[7] Hubel DH, Wiesel TN. Receptive fields, binocular interaction and functional architecture in the cat's visual cortex The Physiological Society. John Wiley & Sons, Ltd; 1962 10.1113/jphysiol.1962.sp006837 PMC1359523 14449617

[8] Reardon S. Rise of robot radiologists Nature news. Nature Publishing Group; 2019 10.1038/d41586-019-03847-z 31853073

[9] Agrawal A, Gans J, Goldfarb A. Prediction machines the simple economics of artificial intelligence Boston, Massachusetts: Harvard Business Review Press; 2018.

[10] Mitchell TM. Machine learning New York, New York: McGraw Hill; 2017.

[11] Sammut C, Webb GI. Encyclopedia of machine learning and data mining New York, New York: Springer Publishing Company; 2017.

[12] Kok JN, Boers E, Kosters WA, et al Artificial intelligence: definition, trends, techniques, and cases. Artif Intell. 2009:1.

[13] Le Berre C, Sandborn WJ, Aridhi S, Devignes MD, Fournier L, Smaïl-Tabbone M, et al Application of artificial intelligence to gastroenterology and hepatology. Gastroenterology. 2019 pii: S0016-5085(14)41412-1. 10.1053/j.gastro.2019.08.058 31593701

[14] Bengio Y. Learning deep architectures for AI. Foundations and trends in Machine Learning. 2009;2 (1):1–127.

[15] Chollet F. Deep Learning with R. Shelter Island, New York: Manning Publications Company; 2018.

[16] Opening the black box of machine learning. Lancet Respir Med. 2018 pii: S2213-2600(18)30425-9. 10.1016/S2213-2600(18)30425-9 30343029

[17] Ford RA, Price WN II. Privacy and accountability in black-box medicine. Michigan Telecommunications and Technology Law Review. 2016 [cited 2020 May 17]. Available from: https://repository.law.umich.edu/cgi/viewcontent.cgi?article=1221&context=mttlr.

[18] Bender E. Unpacking the black box in Artificial Intelligence for medicine. Undark. 2019 [cited 2020 Oct 22]. Available from: https://undark.org/2019/12/04/black-box-artificial-intelligence/

[19] Selvaraju RR, Cogswell M, Das A, Vedantam R, Parikh D, Batra D. Grad-CAM: visual explanations from deep networks via gradient-based localization. arXiv: 1610.02391v4 2019 [cited 2020 May 17]. Available from: https://arxiv.org/pdf/1610.02391.

[20] Sali R, Ehsan L, Kowsari K, Khan M, Moskaluk CA, Syed S, et al CeliacNet: celiac disease severity diagnosis on duodenal histopathological images using deep residual networks. arXiv:1910.03084 2019 [cited 2020 May 17]. Available from: https://arxiv.org/abs/1910.03084. 10.1109/bibm47256.2019.8983270 PMC8740775 35003830

[21] Rajalakshmi R, Subashini R, Anjana RM, Mohan V. Automated diabetic retinopathy detection in smartphone-based fundus photography using artificial intelligence Nature News Nature Publishing Group 2018 10.1038/s41433-018-0064-9 PMC5997766 29520050

[22] Remidio. Home. Available from: https://www.remidio.us/index.php

[23] Eubanks V. Automating inequality: how high-tech tools profile, police, and punish the poor New York, New York: Picador, St. Martin's Press; 2019.

[24] O'Neil C. Weapons of math destruction: how big data increases inequality and threatens democracy London, United Kingdom: Penguin Books; 2018.

[25] Noble SU. Algorithms of oppression: how search engines reinforce racism New York, New York: New York University Press; 2018. 10.1126/science.abm5861 34709921

[26] Privacy in Genomics. National Institutes of Health. National Human Genome Research Institute 2020 [cited 2020 July 6]. Available from: https://www.genome.gov/about-genomics/policy-issues/Privacy.

[27] Big Data to Knowledge. National Institutes of Health. U.S. Department of Health and Human Services. 2020 [cited 2020 May 17]. Available from: http://commonfund.nih.gov/bd2k.

[28] Biomedical Data Science Focus Area Curriculum Requirements. John Hopkins Biomedical Engineering. 2020 [cited 2020 July 6]. Available from: https://www.bme.jhu.edu/graduate/mse/degree-requirements/biomedical-data-science/.

[29] Artificial Intelligence and the Future of Clinical Practice. Massachusetts Medical Society: Artificial Intelligence and the Future of Clinical Practice. 2020 [cited 2020 May 17]. Available from: http://www.massmed.org/Continuing-Education-and-Events/Online-CME/Courses/Artificial-Intelligence/Artificial-Intelligence-and-the-Future-of-Clinical-Practice/.

[30] Intro to AI and Machine Learning: Why all the buzz?. Radiological Society of North America. 2018 [cited 2020 May 17]. Available from: http://education.rsna.org/diweb/catalog/item/eid/1008312688

[31] Biomedical Data Science Curriculum Initiative. Harvard Department of Biomedical Informatics. 2020 [cited 2020 May 17]. Available from: https://dbmi.hms.harvard.edu/education/biomedical-data-science-curriculum-initiative.

[32] Biomedical Data Science Initiative. Stanford Medicine. 2020 [cited 2020 May 17]. Available from: http://med.stanford.edu/bdsi.html

[33] Clarke AC. Profiles of the future: an enquiry into the limits of the possible London, United Kingdom: Pan Books; 1983.

[34] Ross C. IBM’s Watson supercomputer recommended ‘unsafe and incorrect’ cancer treatments, internal documents show. STAT. 2018 [cited 2020 Oct 22]. Available from: https://www.statnews.com/2018/07/25/ibm-watson-recommended-unsafe-incorrect-treatments/

